# Supplementary material for: Drug repurposing for aging research using model organisms
Source: Aging Cell. 2017 Jun 16;16(5):1006–15. doi: 10.1111/acel.12626 (PMC5595691; doi:10.1111/acel.12626)
Supplement: Supplementary file 7 — Data S1 Zip‐Archive of all report cards. [file ACEL-16-1006-s007.zip › RC_FUM.pdf]

FUM

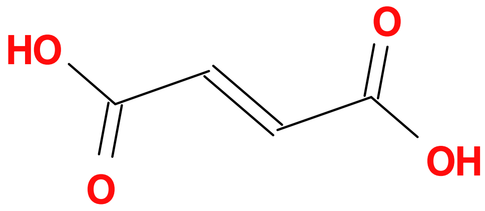

Database identifiers

ChEMBLCompound	CHEMBL503160
DrugBank	DB04299
CHEBI	18012
eMolecules	475652

Ranking

	Rank	Score
Drosophila	412/697	0.349
C. elegans	215/591	0.238

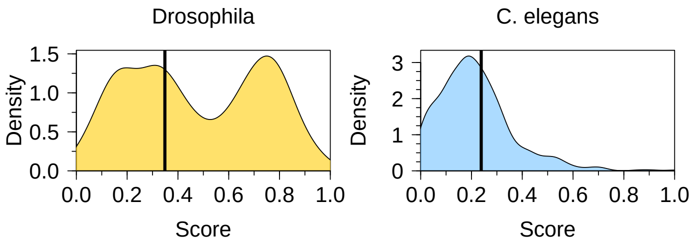

	Ageing implication	Domain conservation	Binding site conservation	Binding affinity	Bioavailability	Lipinski	Promiscuity	Purchasability	Drug approval	**Total**
Drosophila	1.0	0.931	0.738	0.281	(0.9)	0.0	-0.0	0.1	0.075	0.349
C. elegans	1.0	0.938	0.958	0.281	0.253	0.0	-0.0	0.1	0.075	0.238

Names

· **fumaric acid**
· (2E)-2-butenedioic acid
· E297
· Fumarsaeure
· Maleic Acid
· trans-1,2-ethylenedicarboxylic acid
· trans-Butenedioic acid

Roles

fundamental metabolite, food acidity regulator

Status

Approved drug (according to ChEMBL)	No
Classification (according to DrugBank)	experimental
Number of Rule of 5 violations	0
Binding affinity to original target in log units (RF-Score prediction)	4.06
Burns *C. elegans* bioavailability prediction	-1.97

Compound Target Characteristics

NAD-dependent malic enzyme, mitochondrial

Best gene implication in ageing for this target family came from gene Q9NIW0 annotated in UniProt release 2014_02. Annotation GO 8340 (determination of adult lifespan) was Inferred from Mutant Phenotype

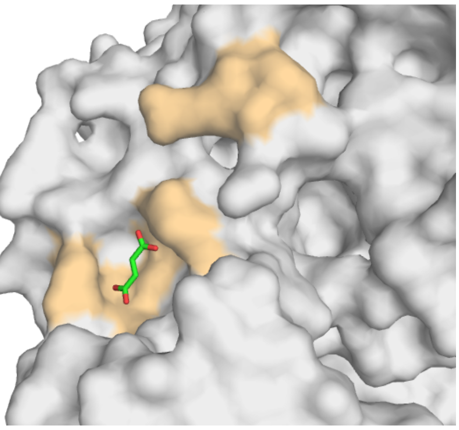

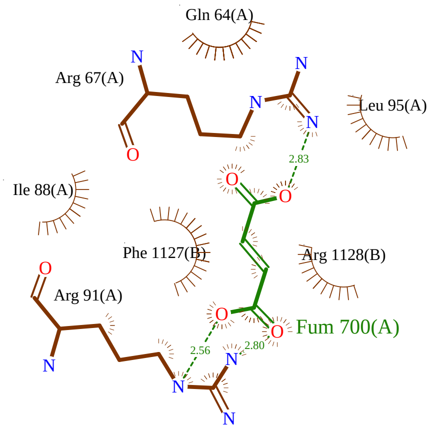

protein	amino acids contacts (binding site)
PDB:1gz3:chainA:P23368	Q R I R L F R
PDB:1gz4:chainA:P23368	Q R I R L F R
PDB:1pj2:chainA:P23368	Q R I R L F R
PDB:1pj4:chainA:P23368	Q R I R L F R
sp:P23368:MAOM_HUMAN	Q R I R L F R
tr:D3ZJH9:D3ZJH9_RAT	Q R I R L F R
tr:Q3V2Y4:Q3V2Y4_MOUSE	Q R I R L F R
sp:Q99KE1:MAOM_MOUSE	Q R I R L F R
tr:H7BWR6:H7BWR6_DROME	Q H L R L H Q
tr:Q9NIW0:Q9NIW0_DROME	Q H L R L H Q
tr:H7BWR4:H7BWR4_DROME	Q H L R L R Q
tr:H7BWU6:H7BWU6_DROME	Q H L R L H Q
tr:H7BWS3:H7BWS3_DROME	Q H L R L H Q
tr:H7BWS2:H7BWS2_DROME	Q H L R L H Q
tr:Q2QBM1:Q2QBM1_DROME	Q H L R L H Q
tr:H7BWS7:H7BWS7_DROME	Q H L R L H Q
tr:H7BWS6:H7BWS6_DROME	Q H L R L H Q
tr:H7BWU8:H7BWU8_DROME	Q H L R L H Q
tr:Q9U1J1:Q9U1J1_DROME	Q H L R L H Q
tr:Q9NIW1:Q9NIW1_DROME	Q H L R L H Q
tr:Q9VG32:Q9VG32_DROME	Q H L R L H Q
tr:Q9NIW2:Q9NIW2_DROME	Q H L R L H Q
tr:Q9VG31:Q9VG31_DROME	Q H L R L H Q
tr:Q9U296:Q9U296_CAEEL	Q R L R L Y R
sp:P36013:MAOM_YEAST	Q R L Q L F R

	whole protein		domain-based		contact-based	
protein	ident	simil	ident	simil	ident	simil
PDB:1gz3:chainA:P23368	0.99	1.0	1.0	1.0	1.0	1.0
PDB:1gz4:chainA:P23368	1.0	1.0	1.0	1.0	1.0	1.0
PDB:1pj2:chainA:P23368	1.0	1.0	1.0	1.0	1.0	1.0
PDB:1pj4:chainA:P23368	1.0	1.0	1.0	1.0	1.0	1.0
sp:P23368:MAOM_HUMAN	1.0	1.0	1.0	1.0	1.0	1.0
tr:D3ZJH9:D3ZJH9_RAT	0.89	0.97	0.96	0.99	1.0	1.0
tr:Q3V2Y4:Q3V2Y4_MOUSE	0.21	0.22	0.42	0.42	1.0	1.0
sp:Q99KE1:MAOM_MOUSE	0.88	0.96	0.96	0.99	1.0	1.0
tr:H7BWR6:H7BWR6_DROME	0.47	0.77	0.54	0.86	0.43	0.73
tr:Q9NIW0:Q9NIW0_DROME	0.46	0.76	0.54	0.86	0.43	0.73
tr:H7BWR4:H7BWR4_DROME	0.46	0.76	0.54	0.86	0.43	0.74
tr:H7BWU6:H7BWU6_DROME	0.45	0.76	0.54	0.86	0.43	0.73
tr:H7BWS3:H7BWS3_DROME	0.46	0.76	0.54	0.86	0.43	0.73
tr:H7BWS2:H7BWS2_DROME	0.46	0.76	0.54	0.86	0.43	0.73
tr:Q2QBM1:Q2QBM1_DROME	0.46	0.76	0.54	0.86	0.43	0.73
tr:H7BWS7:H7BWS7_DROME	0.45	0.76	0.54	0.86	0.43	0.73
tr:H7BWS6:H7BWS6_DROME	0.45	0.76	0.54	0.86	0.43	0.73
tr:H7BWU8:H7BWU8_DROME	0.46	0.76	0.54	0.86	0.43	0.73
tr:Q9U1J1:Q9U1J1_DROME	0.44	0.74	0.54	0.86	0.43	0.73
tr:Q9NIW1:Q9NIW1_DROME	0.36	0.61	0.54	0.86	0.43	0.73
tr:Q9VG32:Q9VG32_DROME	0.36	0.61	0.54	0.86	0.43	0.73
tr:Q9NIW2:Q9NIW2_DROME	0.36	0.61	0.54	0.86	0.43	0.73
tr:Q9VG31:Q9VG31_DROME	0.36	0.61	0.54	0.86	0.43	0.73
tr:Q9U296:Q9U296_CAEEL	0.47	0.78	0.59	0.87	0.71	0.96
sp:P36013:MAOM_YEAST	0.3	0.64	0.4	0.79	0.71	0.93

UniProt:H7BWR6 annotation

Cofactor: Mg(2+) Evidence=(PIRSR:PIRSR000106-3); Name=Mn(2+); Xref=ChEBI:CHEBI:29035; Evidence=(PIRSR:PIRSR000106-3); Note=Divalent metal cations. Prefers magnesium or manganese. (PIRSR:PIRSR000106-3);

(Information from UniProt)

Men (FBgn0002719) associated phenotypes
fast, intermediate, long lived, poor, sleep defective

(Information from FlyBase)

Men (UniProt:Q9NIW0) annotation
Cofactor: Mg(2+) Evidence=(PIRSR:PIRSR000106-3); Name=Mn(2+); Xref=ChEBI:CHEBI:29035; Evidence=(PIRSR:PIRSR000106-3); Note=Divalent metal cations. Prefers magnesium or manganese. (PIRSR:PIRSR000106-3);

(Information from UniProt)

UniProt:H7BWR4 annotation
Cofactor: Mg(2+) Evidence=(PIRSR:PIRSR000106-3); Name=Mn(2+); Xref=ChEBI:CHEBI:29035; Evidence=(PIRSR:PIRSR000106-3); Note=Divalent metal cations. Prefers magnesium or manganese. (PIRSR:PIRSR000106-3);

(Information from UniProt)

UniProt:H7BWU6 annotation
Cofactor: Mg(2+)Name=Mn(2+); Xref=ChEBI:CHEBI:29035; Note=Divalent metal cations. Prefers magnesium or manganese.;

(Information from UniProt)

UniProt:H7BWS3 annotation
Cofactor: Mg(2+) Evidence=(PIRSR:PIRSR000106-3); Name=Mn(2+); Xref=ChEBI:CHEBI:29035; Evidence=(PIRSR:PIRSR000106-3); Note=Divalent metal cations. Prefers magnesium or manganese. (PIRSR:PIRSR000106-3);

(Information from UniProt)

UniProt:H7BWS2 annotation
Cofactor: Mg(2+) Evidence=(PIRSR:PIRSR000106-3); Name=Mn(2+); Xref=ChEBI:CHEBI:29035; Evidence=(PIRSR:PIRSR000106-3); Note=Divalent metal cations. Prefers magnesium or manganese. (PIRSR:PIRSR000106-3);

(Information from UniProt)

Men (UniProt:Q2QBM1) annotation
Cofactor: Mg(2+)Name=Mn(2+); Xref=ChEBI:CHEBI:29035; Note=Divalent metal cations. Prefers magnesium or manganese.;

(Information from UniProt)

UniProt:H7BWS7 annotation
Cofactor: Mg(2+) Evidence=(PIRSR:PIRSR000106-3); Name=Mn(2+); Xref=ChEBI:CHEBI:29035; Evidence=(PIRSR:PIRSR000106-3); Note=Divalent metal cations. Prefers magnesium or manganese. (PIRSR:PIRSR000106-3);

(Information from UniProt)

UniProt:H7BWS6 annotation
Cofactor: Mg(2+) Evidence=(PIRSR:PIRSR000106-3); Name=Mn(2+); Xref=ChEBI:CHEBI:29035; Evidence=(PIRSR:PIRSR000106-3); Note=Divalent metal cations. Prefers magnesium or manganese. (PIRSR:PIRSR000106-3);

(Information from UniProt)

UniProt:H7BWU8 annotation
Cofactor: Mg(2+) Evidence=(PIRSR:PIRSR000106-3); Name=Mn(2+); Xref=ChEBI:CHEBI:29035; Evidence=(PIRSR:PIRSR000106-3); Note=Divalent metal cations. Prefers magnesium or manganese. (PIRSR:PIRSR000106-3);

(Information from UniProt)

Men (UniProt:Q9U1J1) annotation
Cofactor: Mg(2+) Evidence=(PIRSR:PIRSR000106-3); Name=Mn(2+); Xref=ChEBI:CHEBI:29035; Evidence=(PIRSR:PIRSR000106-3); Note=Divalent metal cations. Prefers magnesium or manganese. (PIRSR:PIRSR000106-3);

(Information from UniProt)

men-1 (UniProt:Q9U296) annotation
Cofactor: Mg(2+) Evidence=(PIRSR:PIRSR000106-3); Name=Mn(2+); Xref=ChEBI:CHEBI:29035; Evidence=(PIRSR:PIRSR000106-3); Note=Divalent metal cations. Prefers magnesium or manganese. (PIRSR:PIRSR000106-3);

(Information from UniProt)
